# Why do smokers diagnosed with COPD not quit smoking? - a qualitative study

**DOI:** 10.1186/1617-9625-10-17

**Published:** 2012-10-22

**Authors:** Britt-Marie Eklund, Siv Nilsson, Linnea Hedman, Inger Lindberg

**Affiliations:** 1 The OLIN-studies, Norrbotten County Council, Luleå, Sweden; 2 Department of Health Science, Luleå University of Technology, Luleå, Sweden

**Keywords:** Chronic obstructive pulmonary disease, COPD, Experiences, Smoking cessation, Qualitative content analysis

## Abstract

**Background:**

Chronic Obstructive Pulmonary Disease (COPD) is currently one of the most widespread chronic lung diseases and a growing cause of suffering and mortality worldwide. It is predicted to become the third leading cause of death in the near future. Smoking is the most important risk factor, and about 50% of smokers develop COPD. Smoking cessation is the most important way to improve prognosis. The aim of the study was to describe difficulties of smoking cessation experienced by individuals with COPD who are unable to stop smoking.

**Methods:**

Ten smokers (five women) with COPD, GOLD stage II, participated in semi-structured interviews in 2010. The data were analyzed using qualitative content analysis. The participants were recruited from the Obstructive Lung Disease in Northern Sweden (OLIN) studies.

**Results:**

The participants lives were governed by a lifelong smoking habit that was difficult to break although they had knowledge about the harmful effects and the consequences of COPD. The participants described incidents in their lives as reasons for never finding the time to quit smoking. Demands to quit smoking from other people could lead to continued smoking or get them started again after cessation as they did not want to be patronized. They wanted to receive support from relatives and care providers but they wanted to make the decision to quit on their own.

**Conclusion:**

For successful smoking cessation, it is important to understand the difficulties smokers are experiencing that influence their efforts to quit smoking. To achieve a successful lasting smoking cessation it might be more effective to first ensure that the smoker has the right internal motivation to make the decision to quit, then assist with smoking cessation.

## Background

Chronic Obstructive Pulmonary Disease (COPD) is currently one of the most widespread lung diseases and is a growing cause of suffering and mortality worldwide. It is predicted to become the third leading cause of death in the near future [1]. In northern Sweden, the prevalence of COPD was 14% among people over 45 years of age [2]. Both the prevalence and the incidence increased with age [2,3]. The health economic costs were ten times higher in severe compared to mild COPD, and the authors suggested that early diagnosis is necessary to avoid disease progression and reduce costs for the society [4].

Smoking is the most important risk factor for developing COPD, and about 50% of smokers develop the disease [5]. When diagnosed with COPD, many stop smoking, while some continue to smoke. It is important for smokers with COPD to succeed in smoking cessation before their respiratory health is irreversibly damaged [6]. It has been shown that smoking cessation, even intermittent cessation, reduced the excess lung function decline due to tobacco smoke [7-9], and decreased the risk of exacerbations [10].

COPD is an underdiagnosed disease [3], and obtaining the COPD diagnosis seems important because it has been shown that smoking cessation was more common among those with a diagnosis [11]. Further, studies have shown that long-term behavioural support increased quit rates [12], and that smoking cessation may be more effective when counselling and pharmacological treatments were combined [13]. However, smoking cessation can be difficult to achieve, especially among those with higher nicotine dependence [14,15]. Even after receiving smoking cessation support, COPD patients may not be able to quit smoking [16]. In order to understand why individuals diagnosed with COPD continue to smoke, qualitative studies are required, but very few have been published. One available study showed that having respiratory symptoms was not reason enough to quit, as many of the smokers felt alienated and unworthy of smoking cessation support as they regarded their disease as self-inflicted [17]. The aim of the present study was to describe the difficulties experienced by individuals diagnosed with moderate COPD who are unable to stop smoking.

## Methods

### Study population

A purposive sample of participants in an ongoing case–control study of COPD within the OLIN (Obstructive Lung Diseases in Northern Sweden) studies [18] was invited to participate in a qualitative study using semi-structured interviews in 2010. The participants were selected according to the following inclusion criteria: 50–60 years of age; diagnosed with moderate COPD, i.e., Global Initiative for Chronic Obstructive Lung Disease (GOLD) stage II, with a FEV_1_ (forced expiratory volume) of 50-79% of the expected normal value; have respiratory symptoms including cough and excessive production of phlegm; currently smoking; and to cognitively be able to participate in an interview. A research nurse at the OLIN studies selected 15 participants who fulfilled the criteria and written information about the study was sent by mail. The first five men and five women who responded to the enquiry and gave informed consent were included in the study. One of the researchers (BME) contacted them by telephone and made an appointment for the interview. The study was approved by the ethics committee at Luleå University of Technology.

### Data collection

An interview guide was created by the authors in order to ensure that certain topics regarding smoking cessation was covered (Table 1). The interviews lasted 30–45 minutes, were tape-recorded, and were transcribed by two of the authors (BME and SN). All the interviews were performed at a health care center near the participant’s home according to the patients’ request. The participants were informed that they could withdraw from the study at any time.

**Table 1:** Interview guide for semi-structured interviews about smoking cessation among smokers with chronic obstructive pulmonary disease

**Main questions**	**Follow-up questions**
How long have you been smoking?	
What information did you receive about the benefits of quitting smoking when you were diagnosed with COPD?	
Have you ever tried to quit smoking before you were diagnosed with COPD?	Why did it not work?
Have you ever tried to quit smoking after you have been diagnosed with COPD?	Why did it not work?
If you tried to quit smoking, did you use any aids?	
What are the reasons why you continue to smoke?	
What would help you quit smoking?	
What support would you like to receive if you decided to quit smoking?	
	Can you tell me more?
	Can you please clarify?
	Can you give an example?
	Can you describe what you mean?

### Data analysis

Qualitative content analysis was used to analyse the data [19]. Analysis of the transcribed interview text was conducted in a stepwise manner. First the text was read and divided into meaning units related to the aim, then the units were condensed and coded. Based on the codes’ differences and similarities, they were sorted into subcategories and categories, i.e. the manifest content. The underlying meaning of the interviews was formulated into themes, i.e. the latent content. Several measures to achieve trustworthiness were applied [19,20]. For instance, the transcribed interviews were read several times during the process of analysis so that the context of the data would not be lost and to secure that the data were placed under the right category. During collection and analysis of the data, the researchers had an open dialogue and many steps back and forth were taken between the interview texts and the analysis. In order to increase credibility, quotes from the interviews were presented in the results.

## Results

The interview text was analyzed using qualitative content analysis and resulted in two themes and five categories (Table 2). Quotations from the participants are integrated in the text below in order to emphasize the content.

**Table 2:** Results of qualitative content analyses presented as themes and categories

**Themes**	**Categories**
**Life is governed by a long smoking history that is difficult to break**	- Breaking a lifelong pattern is very difficult
	
	- It is never the right time in life to stop
	
	- Plans to stop do not lead to results
	
**Being aware and enlightened and having a need for autonomy**	- Being aware of the consequences of continued smoking
	
	- To receive help and support without being patronized

### Theme 1. Life is governed by a long smoking history that is difficult to break

This theme describes the difficulty of breaking a habitual behavior. Different circumstances and habits affected the participants’ ability to find an appropriate time to stop smoking. Smoking was associated with specific events and feelings that gave positive as well as negative experiences, which made it even more difficult to quit smoking.

#### Breaking a lifelong pattern is very difficult

The participants had begun smoking when they were 12 to 13 years old. Most of them saw a connection between smoking and their life patterns. Smoking was perceived to give pleasure which was strongly associated with specific situations, such as smoking during meals, and coffee or alcohol intake. Such pleasure was a positive experience in their life, and it was an very difficult habit to break. Some of the participants described that smoking was a reward in life, for instance, after hard work. Half of the participants described that the cigarette was a companion and dear friend, even best friend, and a part of their community. The habit of holding something between the fingers made them light a cigarette even though there was no craving to smoke.

“It’s in the morning with coffee… the first two cigarettes… That’s great.”

#### It is never the right time in life to stop

The participants described incidents in their lives as reasons for never finding the time to focus on smoking cessation. Their hectic everyday life led to continued smoking, although smoking was not always something they longed to do. Some participants felt that the cigarette was comforting and suppressed worries. Other participants felt that the stress associated with the illness or death of a relative justified that they could not stop smoking, and so the occasion to quit was postponed.

“First my sister-in-law died, four weeks later my husband died, and four months later my dad died.”

Although the participants realized the benefits of quitting smoking, they expected life without smoking to be ascetic. The participants had often thought about giving up smoking but always as something to be done in the future; now is not the right time.

“we discuss, my brother, my sister and her daughter to agree on a date when we must … but all slip on that … on date … we’ll see.”

Some of the participants had stopped smoking but had experienced weight gain, which led them to start smoking again.

“Yes, I’m just saying if I would not put on extra weight then I would certainly make an effort to try to quit, but it’s the weight I’m afraid of.”

Other reasons that were given for not quitting were the risks of dizziness, hypotension, or depression.

#### Plans to stop do not lead to results

Several of the participants expressed that the reason why it was impossible to quit smoking was “all in the brain.” They had a hard time explaining what they really meant by this, one participant said:

“Perhaps one has to get lobotomized … it is in the brain.’

Some of the participants seemed to have control over most things in their life, except smoking. They considered their being addicted to the cigarette as a scourge, which led to their plans to stop smoking never becoming realized. The participants realized that it was necessary to have the motivation to quit smoking, but such motivation was missing, and their plans to quit did not lead to any results.

“I would like to have a verdict … if you don’t stop smoking, you will die now or in a year … a slap in the face.”

Having close relatives with poor lung capacity and the knowledge that smoking is connected with high costs, impaired physical condition, or even death increased their motivation to quit, but even these were not enough. Half of the participants had decreased their smoking but had not been able to stop completely.

“So I have smoked, perhaps twenty cigarettes a day earlier and now maybe four … so I’ve reduced it over a long time, so to speak.”

Some of them believed that it is easy to refrain from smoking when being in specific environments, doing specific chores, or in areas where smoking is forbidden. It was important to remove distractions, to be in control, and to have peace and quiet. Furthermore, while there were positive factors that contributed to a smoking cessation plan, such as if nobody was smoking in their environment, if cigarettes were not available, various activities, travel, and exercise, plans to quit entirely were never accomplished.

“One day, I will make the decision … but it has been that way for many years … in terms of reasoning, and darn it …I am still smoking.”

### Theme 2. Being aware and enlightened and having a need for autonomy

This theme describes an awareness of the risks of smoking and the consequences of COPD. It was difficult to deal with the surrounding demands of smoking cessation since the decisions had to be taken independently in order to keep their autonomy. Support should be given after the individual has made his or her own decision.

#### Being aware of the consequences of continued smoking

The participants were aware of the consequences of continued smoking and had knowledge of COPD. They knew that people with the disease never regained their health but that the progression of the disease halted after smoking cessation. The annual lung function test performed within their participation in the OLIN studies showed the extent of the disease. For some participants, it was a good help to start thinking about quitting, while others felt that it was not important. For a number of participants, the lung function test showed beyond all doubt that they needed to make the decision to quit smoking.

“To get information about lung function impairment … that’s when one got the feeling … now I really have to fix it.”

“My father-in-law has lung cancer and is dying, and my mom had COPD and had no good days at the end of her life … so I know what it’s like … for no use.”

#### *To receive help and support without being patronized*

Some participants wanted help in the same way that alcoholics were given help, such as through a twelve-step program. Other participants said that support was needed after setbacks.

“That one would get support because there will be a big downfall and especially if there is any adversity that I run into…then you feel like… No, I do not give a damn.”

The best support was found among relatives. It was easier to be open and talk to their loved ones because of the close relation. Several of the participants felt that it would be easier to quit smoking if their smoking relatives also thought about quitting.

“I probably have the best support among my friends … I think so anyhow.”

The participants wanted help and support, but they did not want to be patronized. The participants experienced demands about smoking cessation, in some cases daily, from spouses, family, friends, employers, and physicians. Nagging from people in their environment could lead to continued smoking or get them started again after smoking cessation.

“When they say…shouldn’t you stop smoking?… never… just because they say so…one thinks never.”

Quitting smoking was a personal choice, and therefore it was the person who decided when or if smoking cessation would happen. Some of the participants experienced having people in their environment who were ignorant of their situation. Telling someone about their situation could result in experiencing pressure to quit, so the participants had not informed anyone about quitting smoking.

“Then I think to myself … this is none of your business … it’s my own choice.”

Participants described that while smoking was previously fully accepted and considered fashionable, nowadays it is no longer so. In fact, smoking can be considered as a weakness, and being a smoker can be regarded as bad as being an alcoholic. Smoking was considered shameful since the act is prohibited in public places in Sweden.

“That in recent years I have felt myself being chased by a blowtorch,and the kids think we are totally worthless because we smoke.”

Almost all the participants had tried smoking cessation drugs of various kinds. Their experiences with treatments were both positive and negative. Several of the participants said they had experienced side effects that made them cancel the treatment.

“I’ve tried to use plasters … and then I felt … that I became dizzy … dizzy.”

Some participants used snus (Swedish moist ground tobacco placed under the upper lip) as a substitute for cigarettes. Several participants had experienced temporary help from drugs. Some had been involved in smoking cessation groups at the medical center, but they did not think that group meetings were helpful because all the participants in the group had not decided to quit smoking. Furthermore, one participant who had attempted smoking cessation said that it was easier to refrain from smoking as long as he was involved in the weaning group. In earlier attempts to quit, several participants had not used any aids. Almost all the participants were critical of the information and support they had gotten from health care professionals.

## Discussion

Smoking cessation is the most important intervention to reduce the risk for cardiovascular and respiratory diseases [6], especially COPD. While most individuals understand the benefits of smoking cessation and many who are diagnosed with COPD quit smoking, some continue to smoke. In order to understand why individuals diagnosed with COPD continue to smoke, qualitative studies are required. The purpose of the present study was to describe the difficulties of smoking cessation experienced by individuals diagnosed with moderate COPD who had been unable to stop smoking. We have shown that the participants had a long habit of smoking; they had begun smoking when they were between 12 and 13 years old. The participants’ lives were governed by a lifelong smoking habit that was difficult to break although they had knowledge about the harmful effects of smoking. The participants described incidents in their lives as reasons for never finding the time to focus on smoking cessation. Motivation and support are needed after the smoker has made the decision to quit. Those participants who hesitated to make the decision also felt critical of the information and support that were provided. Support should be given after the individual has made his or her own decision. The person’s autonomy must be ensured, and respect for his or her sovereignty must be given.

Because nicotine dependence is a strong addiction and smoking is related to a feeling of pleasure, smoking often becomes a lifelong habit. It has been reported that already at a young age, 14–17 years, smokers experience a strong urge to smoke [21]. Similar to the experiences described by the participants in the present study, smoking initiation at a young age has been shown to be related to a lifelong dependence on nicotine [22]. The feeling of pleasure whenever they smoked was described as a positive experience in their lives. This finding is supported by other studies that have shown that smokers often smoked after a meal, when they had coffee, during a break, while drinking alcohol, and when socializing with other people such as co-workers [23]. Besides the feeling of pleasure, another factor that made the participants want to smoke more was stress and pressure at work, which is in accordance with a study by Kouvonen et al. [24].

The participants in the present study had plans to quit smoking, but these had not been actualized. Other stressful situations in life, such as having ill relatives, occasions of death in the family, or depression, were some of the reasons that led to difficulties in finding the right time to quit smoking. As in other studies [25], weight gain was another reason to start smoking again after cessation for some of the participants. This was not a surprising result since most smokers know that nicotine dependence is a strong addiction and that smoking cessation entails substantial behavior modification that requires a huge effort. Therefore, the decision to quit smoking is postponed. Both the reasons for not quitting, and the reasons leading up to the decision to quit smoking, vary among individuals. Health care professionals should be aware of and take these individualities, as well as the smokers’ motivation to quit, into account in providing smoking cessation support [26]. Further, it is important to understand that smoking cessation is not a single problem to be solved; support regarding other circumstances in the smoker’s life, such as weight gain, stress, and depression, should also be included in order to achieve successful smoking cessation. It has been shown that persons with COPD are more likely to develop depression and anxiety [27,28]. Several of our participants described that they began to smoke again after smoking cessation because of depression, which is in accordance with other studies [29,30].

The participants were aware of the consequences of continued smoking. Some wished to continually receive information about their decreasing lung function or even get a verdict on whether they would die if they continued to smoke. While some studies have shown that worries about future health problems motivated smokers to achieve smoking cessation [31,32], another study showed that having unpleasant respiratory symptoms were not enough [17]. The chart by Fletcher et al. [8] can be used to make decreases in lung function apparent and thereby motivate smokers to quit at an earlier age. However, some smokers find the available smoking cessation support and information insignificant. One study showed that half of the smokers quit spontaneously without any support or planning [33]. All the participants in the present study had been informed about the available support from the health care system, but not all had used it. In another study, smokers described that they were not interested in joining support groups because they expected these to be ineffective [34]. This is in contrast to the finding in the present study that while the participants did not want support before they have made the decision to quit, they wanted support after they have decided to quit. The participants in the present study described that friends and relatives expressed their wish for them to stop smoking, but this had the opposite result. It is thus important that smokers maintain their autonomy; no one can make the decision to quit smoking for them. May et al. reported that in the long term, personal factors, such as self-confidence and nicotine dependence, play a stronger role in successful smoking cessation than social support [35]. The important issue for successful smoking cessation seems to be the understanding of individual differences. Health professionals involved in smoking cessation support should recognize the individual smoker in his or her full life situation and adapt the support thereafter.

The strength of this study design is the possibility to give a further understanding of why individuals with COPD continue to smoke, which would not be possible in a quantitative study. One limitation of the study could be the sample size as more participants might have yielded a different result. However, there are no rules for sample size in qualitative research, but six to eight participants can be sufficient when the sample are a homogenous group [36]. In the present study, the participants were selected homogenously based on age, COPD diagnosis and severity, respiratory symptoms and smoking status. Further, the sample should be based on informational needs and data saturation [37]. The interviews in the present study provided rich and deep content, and although the small sample size does not allow generalization, the results can be transferred to other settings and be useful for health care personnel involved in smoking cessation work. The interviews were performed at a health care center, which might have made the participants feel uncomfortable. However, the participants were asked if they wanted to be interviewed at home, but none wanted to.

In conclusion, the participants’ lives were governed by a lifelong smoking habit that was difficult to break although they had knowledge about the harmful effects of smoking. Plans to quit were never actualized despite being diagnosed with COPD. The smokers described incidents in their lives as reasons for never finding the time to focus on smoking cessation. In order to help smokers with COPD to quit smoking, health professionals involved in smoking cessation support should recognize individual smokers in their full life situation and adapt the support thereafter. To achieve a successful lasting smoking cessation it might be more effective to first ensure that the smoker has the right internal motivation to make the decision to quit, then assist with smoking cessation.

## Competing interest

None of the authors have any conflict of interest to disclose.

## Authors’ contributions

BME designed the study, collected and analyzed data, and drafted the manuscript. SN designed the study, analyzed data and helped to draft the manuscript. LH drafted the manuscript. IL designed the study and helped to draft the manuscript. All authors approved the final manuscript.
